# Supplementary material for: Promoting subjective preferences in simple economic choices during nap
Source: eLife. 2018 Dec 6;7:e40583. doi: 10.7554/eLife.40583 (PMC6294547; doi:10.7554/eLife.40583)
Supplement: Supplementary file 1. — (A) Table 1A. Subject information. All participants were asked to report their subjective level of hunger (from 1 = ‘not hungry at all’ to 8 = ‘very hungry’) and vigilance (from 1 = ‘very vigilant, not sleepy at all’ to 7 = ‘very sleepy, taking great efforts to keep awake’) before and after the verbal stimulation. Parentheses contain standard deviations. p-Values are calculated using two-tailed Student T test for comparing the difference in mean of each variable between the sleep and wake groups. Parentheses contain standard deviations. (B) Table 1B. Snack items included in the study together with their English translations. Snack items were selected based on a pilot experiment in which 49 subjects were recruited to assess the familiarity, valence, and subjective arousal (Self-Assessment-Manikin scale) of a pool of candidate snacks. Based on those ratings, we selected 60 items with median familiarity (mean ±SD: 3.51 ± 0.84), positive valence (mean ±SD: 5.08 ± 1.26), and median arousal level (mean ±SD: 4.65 ± 1.33) (associated with Figure 1). (C) Table 1C. Linear regressions on the effect of verbal cueing for sleep and wake groups after controlling for differences in age, gender, BMI, as well as self-reported familiarity and differences in vigilance and hunger before and after the cueing session. The dependent variable in the first regression is equal to the average difference of ΔWTP between cued and uncued items for each subject. The dependent variable in the second regression is equal to the likelihood of choosing cued over uncued item in the binary decision task for each subject. *p < 0.05; **p < 0.01; ***p < 0.001, two-tailed (associated with Figure 2). (D) Table 1D. Durations of sleep stages (in minutes) for sleep group subjects with or without ERP. There is no significant difference in the duration of sleep stages. Parentheses contain standard errors. p-Values were calculated using two-tailed Student T test for between-group comparisons (associated with Figure [file elife-40583-supp1.docx]

**Supplementary file 1:** **Supplementary Tables 1A through 1I**

**Supplementary file 1. Table 1A.**

|  | Sleep | Wake | P value |
| --- | --- | --- | --- |
| #Subjects | 47 | 45 |  |
| Gender | 40 F | 39 F |  |
| Age | 23.43 (2.38) | 23.42 (2.37) | 0.29 |
| BMI | 20.64 (2.06) | 20.38 (2.19) | 0.07 |
| Pre-stimulation hunger | 3.93 (1.48) | 4.16 (1.72) | 0.51 |
| Post-stimulation hunger | 4.79 (1.96) | 5.66 (1.72) | 0.03 |
| Pre-stimulation vigilance | 3.79 (1.08) | 3.80 (1.42) | 0.96 |
| Post-stimulation vigilance | 2.21 (1.10) | 2.76 (1.68) | 0.07 |
| Hunger (post-pre) | 0.83 (2.22) | 1.50 (1.98) | 0.13 |
| Vigilance (post-pre) | -1.57 (1.47) | -1.04 (1.97) | 0.14 |

**Supplementary file 1. Table 1B.**

| 士力架 | Snickers | 彩虹糖 | Skittles |
| --- | --- | --- | --- |
| 德芙巧克力 | Dove chocolate | **百奇** | Pocky |
| MM花生豆 | M&M Peanut | **奥利奥** | Oreos |
| 趣多多 | Chips ahoy | **乐事经典** | Lays classic |
| 乐天饼 | Lotte biscuits | **上好佳薯片** | Oishi |
| 好时巧克力 | Hershey chocolate | **旺旺雪饼** | WantWant crackers |
| 盼盼面包 | Pan-Pan bread | **小熊饼干** | Teddy crackers |
| 奇多妙脆角 | Cheetos bugles | **雀巢脆脆鲨** | Nestle wafer |
| 阿尔卑斯糖 | Alpenliebe candy | **金帝巧克力** | Leconte chocolate |
| 旺仔牛奶糖 | Hot-kid’s nougat | **百力滋** | Pretz |
| 亲亲虾条 | Qinqin prawn cracker | **康师傅饼干** | Masterkong biscuits |
| 品客薯片 | Pringles potato chips | **可比克** | Copico |
| 达利园面包 | Dali bread | **好丽友派** | Orion pie |
| 提拉米苏 | Tiramisù | **百醇** | Pejoy |
| 费列罗巧克力 | Ferrero chocolate | **泡芙** | Puff |
| 太平苏打饼 | Pacific soda cake | **MM巧克力豆** | Chocolate MM |
| 沙琪玛 | Caramel treats | **多利多兹** | Doritos |
| 米老头 | Uncle pop | **蔓越莓饼** | Cranberry cookies |
| Q蒂 | Qute | **港荣蛋糕** | Kongweng cake |
| 妙芙蛋糕 | Muffin cake | **小浣熊面** | Small raccoon noodle |
| 金丝猴糖 | Goldenmonkey candy | **大白兔糖** | White rabbit candy |
| 奇趣蛋 | Kinder joy | **奇巧** | KitKat |
| 杏仁巧克力 | Almond chocolate | **乐之饼干** | Ritz biscuits |
| 焙朗饼干 | Belvita biscuits | **波力卷** | Bonny roll |
| 麦吉士爆米花 | Mage’s popcorn | **瑞士莲** | Lindt chocolate |
| 蘑菇力 | Moguli chocolate | **老奶奶花生米** | Grandma peanut |
| 康师傅苏打饼 | Masterkong soda cake | **小王子雪饼** | Little prince rice cake |
| 黄飞红花生米 | Huangfeihong peanut | **达利园派** | Daliyuan pie |
| 华夫饼 | Waffle | **燕麦糖** | Oatmeal candy |
| 苹果派 | Apple pie | **瑞士卷** | Swiss roll |

**Supplementary file 1. Table 1C.**

|  | ΔWTP_cued_ - ΔWTP_uncued_ | | % choosing cued item | |
| --- | --- | --- | --- | --- |
| Variable | β | P value | β | P value |
| Intercept | 0.4975 | 0.6678 | 0.7583 | 0.0016** |
| Treatment (sleep =1) | 0.3946 | 0.0095** | 0.0707 | 0.0206* |
| Vigilance (post − pre) | - 0.0639 | 0.1470 | - 0.0062 | 0.4832 |
| Hunger (post – pre) | 0.0640 | 0.0774 | 0.0071 | 0.3329 |
| Familiarity | 0.0487 | 0.3604 | 0.0128 | 0.2346 |
| BMI | - 0.0237 | 0.5339 | - 0.0064 | 0.4011 |
| Age | - 0.0078 | 0.8053 | - 0.0071 | 0.2469 |
| Gender (Male = 1) | 0.1394 | 0.5541 | 0.0708 | 0.1384 |
| R^2^ | 0.1418 | | 0.1362 | |
| # Observations | 92 | | 92 | |

**Supplementary file 1. Table 1D.**

|  | Sleep group  (PSG only) | Sleep group  (PSG & ERP) | P value |
| --- | --- | --- | --- |
| #Subjects | 24 | 23 |  |
| Sleep latency | 11.25 (1.64) | 10.04 (1.27) | 0.56 |
| N1 | 14.06 (1.92) | 12.93 (1.94) | 0.68 |
| N2 | 31.90 (2.77) | 33.17 (2.84) | 0.75 |
| N3 | 18.63 (2.54) | 19.54 (3.14) | 0.82 |
| REM | 7.00 (1.88) | 4.89 (1.62) | 0.40 |
| Total sleep time | 71.58 (4.10) | 70.54 (3.43) | 0.85 |

**Supplementary file 1. Table 1E.**

|  | Sleep group  (PSG only) | Sleep group  (PSG & ERP) | P value |
| --- | --- | --- | --- |
| #Subjects | 24 | 23 |  |
| ∆WTP_cued_-∆WTP_uncued_ | 0.560 (0.135) | 0.582 (0.133) | 0.91 |
| WTP1_cued_-WTP1_uncued_ | 0.018 (0.025) | 0.000 (0.018) | 0.56 |
| WTP2_cued_-WTP2_uncued_ | 0.578 (0.130) | 0.581 (0.132) | 0.99 |
| % choosing cued items | 60.42 (1.67) | 58.42 (2.82) | 0.54 |

**Supplementary file 1. Table 1F.**

|  | KC + | | | KC - | | |
| --- | --- | --- | --- | --- | --- | --- |
| Electrodes | Mean | SD | P value | Mean | SD | P value |
| F3 | 3.5066 | 4.6899 | 0.0099*** | -6.3904 | 4.3861 | 0.0000*** |
| F4 | 3.1662 | 3.9324 | 0.0051** | -5.7372 | 4.2025 | 0.0000*** |
| C3 | 2.4462 | 3.1824 | 0.0078** | -4.3022 | 2.9252 | 0.0000*** |
| C4 | 2.3274 | 3.1161 | 0.0010** | -4.1318 | 3.0989 | 0.0000*** |
| O1 | -0.0864 | 0.7868 | 1 | -0.3093 | 0.8755 | 0.6258 |
| O2 | 0.1819 | 0.6736 | 1 | -0.7591 | 1.2755 | 0.0554 |

**Supplementary file 1. Table 1G.**

|  | Across subjects | | Across cued items | |
| --- | --- | --- | --- | --- |
|  | r | P value | r | P value |
| α [8-12Hz] | 0.3589 | 0.4632 | 0.2928 | 0.2290 |
| β [12-30Hz] | 0.2465 | 1 | 0.2011 | 0.8765 |
| θ [4-8Hz] | 0.6409 | 0.0049** | 0.4667 | 0.0047** |
| δ [0-4Hz] | 0.6527 | 0.0037** | 0.5160 | 0.0010** |
| γ[30-35Hz] | -0.0197 | 1 | -0.0230 | 1 |
| # Observations | 23 | | 47 | |

**Supplementary file 1. Table 1H.**

| Parameter | Level | Prior |
| --- | --- | --- |
| Mean drift rates for cued | individual | *k_cued_ ~ Uniform (0, B)* |
| Mean drift rates for uncued | individual | *k_uncued_ ~ Uniform (0, B)* |
| Starting-point variability | individual | *A* ~ 12 ×*Beta* (α_A_, β_A_) |
| Decision threshold | individual | *b* ~ 5×*beta* (α_d_, β_d_) + A |
| Non-decision time | individual | *NDT* ~ *Beta* (α_NDT_, β_NDT_) |
| B | group | *B ~ Normal (5, 0.5)* |
| α_d_ | group | α_d_ ~ *Uniform* (1, 5) |
| α_A_ | group | α_A_ ~ *Uniform* (1, 5) |
| α_NDT_ | group | α_NDT_ ~ *Uniform* (1, 5) |
| β_d_ | group | β_d_ ~ *Uniform* (1, 5) |
| β_A_ | group | β_A_ ~ *Uniform* (1, 5) |
| β_𝜏_ | group | β_𝜏_ ~ *Uniform* (1, 5) |

**Supplementary file 1. Table 1I.**

|  | **Simulated RT distributions** | | | | **Actual RT distributions** | | | |
| --- | --- | --- | --- | --- | --- | --- | --- | --- |
| **Percentile** | **Cued**  **High** | **Cued**  **Low** | **Uncued**  **High** | **Uncued**  **Low** | **Cued**  **High** | **Cued**  **Low** | **Uncued**  **High** | **Uncued**  **Low** |
| 10% | 1.10 | 1.18 | 1.11 | 1.17 | 1.13 | 1.21 | 1.10 | 1.16 |
| 30% | 1.38 | 1.48 | 1.35 | 1.47 | 1.36 | 1.52 | 1.33 | 1.45 |
| 50% | 1.65 | 1.77 | 1.60 | 1.77 | 1.66 | 1.76 | 1.62 | 1.79 |
| 70% | 1.97 | 2.13 | 1.91 | 2.13 | 1.94 | 2.21 | 1.89 | 2.15 |
| 90% | 2.54 | 2.77 | 2.50 | 2.77 | 2.88 | 2.89 | 2.24 | 2.90 |
